# Fatal Itching and Failing Liver: A Case Report and Literature Review of Rare, Atypical DRESS (Drug Rash with Eosinophilia and Systemic Symptoms) Syndrome

**DOI:** 10.7759/cureus.55355

**Published:** 2024-03-01

**Authors:** Mohammed Abusuliman, Mahmoud Amreia, Sheema Rehman, Ammad J Chaudhary, Hazem Abosheaishaa, Taher Jamali, Amir Hanafi

**Affiliations:** 1 Internal Medicine, Henry Ford Health System, Detroit, USA; 2 Internal Medicine, Rochester Regional Health, Rochester, USA; 3 Internal Medicine, Icahn School of Medicine at Mount Sinai, Queens Hospital Center, New York City, USA; 4 Internal Medicine/Gastroenterology, Cairo University, Cairo, EGY; 5 Gastroenterology, Henry Ford Health System, Detroit, USA

**Keywords:** allopurinol hypersensitivity, drug hypersensitivity reaction, drug reaction with eosinophilia and systemic symptoms, renal failure, liver failure, drug reaction with eosinophilia and systemic symptoms (dress) syndrome

## Abstract

DRESS (Drug Rash with Eosinophilia and Systemic Symptoms) syndrome is a rare, life-threatening, hypersensitivity reaction. The prolonged course and non-specific symptoms of the condition make diagnosis challenging. We present a case of DRESS syndrome that was misdiagnosed as urticaria. Investigations revealed deranged liver and kidney functions and abnormal blood count. The presented case emphasizes the need to have a high suspicion for DRESS syndrome in patients who present with jaundice, generalized rash, acute renal failure, and acute liver failure.

## Introduction

DRESS (Drug Rash with Eosinophilia and Systemic Symptoms) syndrome is a severe, potentially fatal, idiosyncratic drug reaction [1]. The overall incidence of DRESS syndrome is not common and is estimated to occur in 0.9-2 per 100,000 patients per year [2], with the estimated risk being one in 1,000 to one in 10,000 after drug exposure [3]. However, the mortality rate approaches 10% in adults [4]. 

The etiology of the syndrome is not well understood, but it is believed to occur as a result of complex interactions between different biological and environmental factors [5]. The disease course is often prolonged, and the clinical presentation is varied with flares occurring even after cessation of the offending drug [1]. 

The syndrome is characterized by extensive skin rash, eosinophilia, atypical lymphocytosis, and organ involvement [6]. Clinical manifestations are not immediate and typically appear within two to eight weeks after the introduction of the triggering drug [7]. The most common systemic involvement is hepatitis followed by nephritis, pneumonitis, myositis, and gastroenteritis [8]. Cholestasis can also occur although rarely [9]. 

The most common drugs triggering DRESS syndrome are allopurinol, aromatic antiepileptic agents, sulfonamides, vancomycin, minocycline, nevirapine, antituberculosis agents, and mexiletine [10]. Most cases of DRESS syndrome resolve over the course of two to three months after stopping the offending agent; however, some cases progress to multiple organ affection. 

## Case presentation

**Figure 1:**
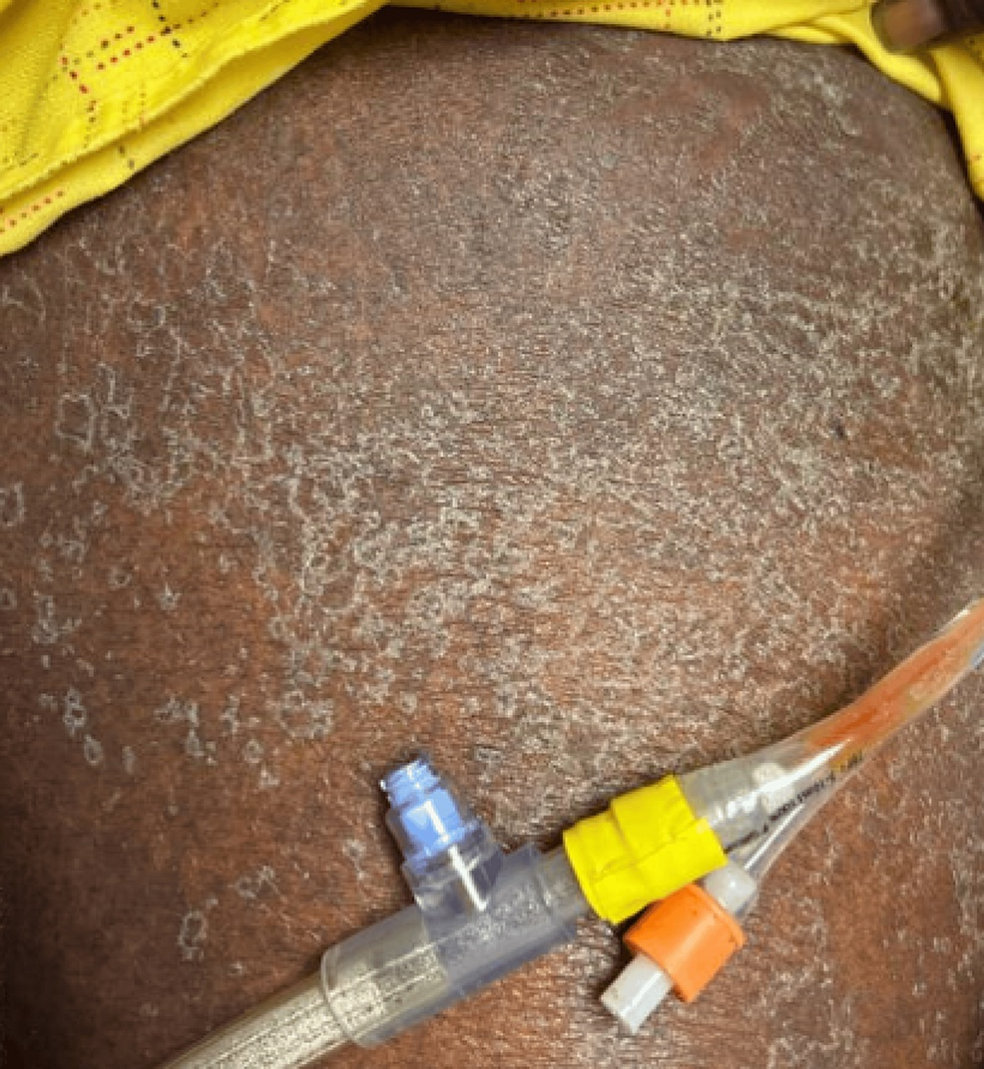
Scaly skin visible at presentation The patient complained of itching and scaly skin. She was prescribed levocetirizine and was initially misdiagnosed as idiopathic urticaria. Although the itchiness resolved, her skin became scalier.

A 55-year-old female with a past medical history significant for hypertension, gout, and chronic kidney disease (CKD) stage 3 presented with worsening itching and weakness. She had developed an episode of gout two months earlier for which she had been prescribed allopurinol. She then developed generalized itching and was prescribed prednisone which seemed to help with her symptoms. She was seen by an allergist who prescribed levocetirizine and was initially misdiagnosed as idiopathic urticaria. She continued to take allopurinol and, although the itchiness resolved, her skin became scalier (Figure 1). Her husband noted that she developed yellowish discoloration of the eyes with dark urine and light-colored stool associated with generalized weakness and fatigue. 

**Figure 2:**
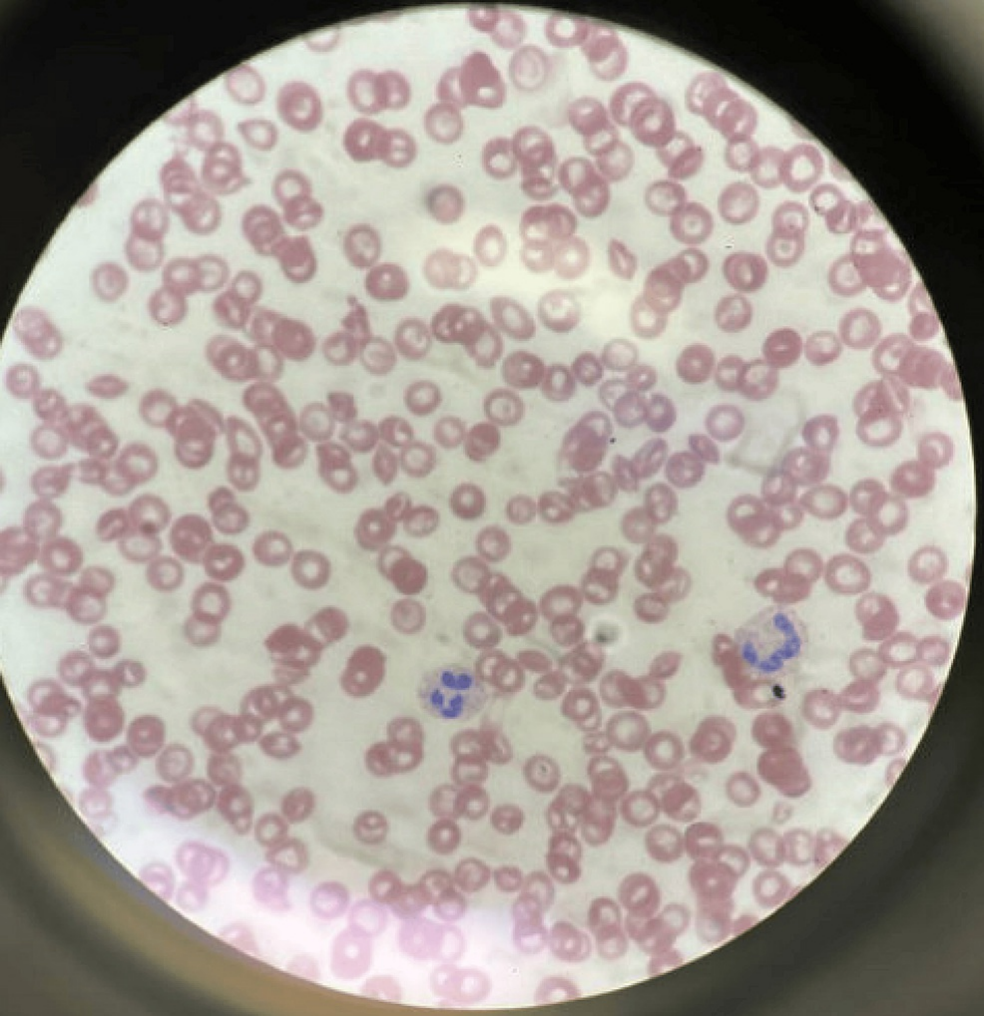
Peripheral blood smear showing thrombocytopenia

On investigation, she was found to have acute renal failure, cholestatic jaundice, and thrombocytopenia (Figure 2) with peripheral blood smear showing no visible platelets.

Her admission lab investigations (Table 1) showed a total bilirubin of 4.5 mg/dl, direct bilirubin of 3.7 mg/dl, aspartate aminotransferase (AST) of 592 U/L, alanine transaminase (ALT) 1130 U/L, creatinine 4.3 mg/dL, INR 1.9. Her platelets were initially 127 K.

**Table 1:** Laboratory investigations at admission

Test	Result	Reference ranges
Hemoglobin	8.2 g/dl	11.5-16 g/dl
RBCs	3 x 10^6^/ul	3.8-5.2 x10^6^/ul
Hematocrit	23%	40-52%
Mean corpuscular volume	75 fl	81–99 fl
Mean corpuscular hemoglobin	26 pg	26–34 pg
Mean corpuscular haemoglobin concentration	36/dl	30–63 g/dl
Red cell distribution width	19.4%	11.5-15%
Platelets	127x 10^3^/ul	140 –400 x 10^3^/ul
WBCs	9.8 x 10^3^/ul	4-10 x 10^3^/ul
Alanine transaminase	1130 U/L	7–55 U/L
Aspartate aminotransferase	592 U/L	8 –48 U/L
Total bilirubin	4.5 mg/dl	0.1–1.2 mg/dl
Direct bilirubin	3.7 mg/dl	<0.3 mg/dl
International normalized ratio	1.9	<= 1.1
Serum creatinine	4.3 mg/dl	0.7–1.3 mg/dl

**Figure 3:**
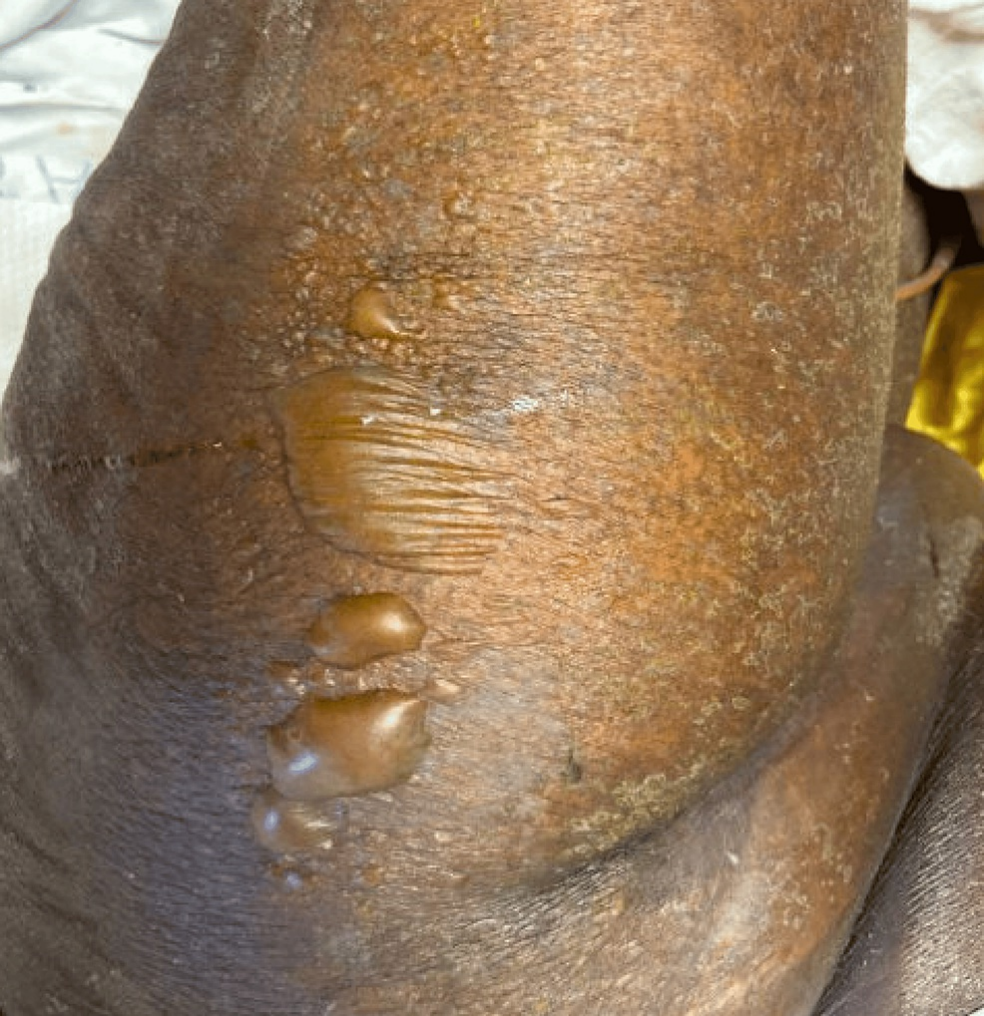
Bullous lesions on the patient's left hip Biopsy of lesion was consistent with cutaneous drug reaction

Hepatitis B and C were negative, and she had positive hepatitis A IgG antibodies. The allopurinol was stopped and she underwent several imaging including CT scan of the abdomen and magnetic resonance cholangiopancreatography (MRCP), which did not show any obstructing mass. Lesions on the left hip were bullous (figure 3) and a skin biopsy of lesions showed subacute interface and perivascular dermatitis with prominent dyskeratosis and subepidermal splits mostly consistent with a drug reaction. The working diagnosis was Stevens-Johnson Syndrome (SJS) and toxic epidermal necrolysis (TEN), and she was started on 1 mg/kg of prednisone. Dermatology determined that her thrombocytopenia was likely due to immune thrombocytopenic purpura (ITP). She was started on IV Decadron.

The hospital course was complicated by *Staphylococcus aureus* bacteremia treated with cefazolin, then vancomycin-resistant *Enterococcus* (VRE) bacteremia treated with daptomycin, and herpes simplex virus sacral ulcers. She developed worsening encephalopathy, hypothermia, left shift, and rising lactate requiring her to be transferred to the medical intensive care unit (MICU). Her overall picture was complicated by acute respiratory distress syndrome (ARDS) requiring intubation as well as resistant septic shock. She was shifted to comfort care and eventually passed away. 

## Discussion

DRESS syndrome, also termed drug-induced hypersensitivity syndrome (DIHS) and drug hypersensitivity syndrome (HSS), is a severe adverse drug reaction. With the introduction of hydantoin as an antiepileptic drug in the 1940s, the first instances of DRESS syndrome were reported as lymphadenopathy (LAD) [11]. Lymph node biopsy revealed a lymphomatous appearance, which was then termed drug-induced pseudolymphoma by Satlztein et al. [12]. 

The introduction of another antiepileptic drug, carbamazepine, was followed by reports of a reaction consisting of rash, fever, and LAD. The reaction was then termed anticonvulsant hypersensitivity syndrome (AHS) [13]. Shortly thereafter, several medications were found to exhibit a similar spectrum of side effects, thus the term DIHS, also known as HSS, was coined. The term "DRESS syndrome" was first coined by Bocquet et al. [8], based on the findings of a study by Callot et al. [14]. Schlienger et al. suggested another term: drug-induced delayed multiorgan hypersensitivity syndrome (DIDMOHS) [15]. 

According to estimates, 0.9-2 patients out of every 100,000 patients experience DRESS annually [2]. In hospitalized patients, it represents 10-20% of all cutaneous adverse drug reactions [16]. The risk of developing DRESS varies depending on the medication. The incidence of DRESS for high-risk anticonvulsant drugs is estimated to be one in 1000 to one in 10,000 exposures [17]. 

Drug exposure is one of the main risk factors for developing DRESS, and in 80% of cases, a distinct drug trigger may be found. Drug exposure is not proven in 2% of instances, and in the remaining 10-20% of cases, the drug causality is less obvious [18]. The most common drugs triggering DRESS syndrome are aromatic antiepileptic agents, allopurinol, sulfonamides, vancomycin, minocycline, nevirapine, antituberculosis agents, and mexiletine [10]. 

Another risk factor is pharmacogenetic susceptibility. Numerous human leukocyte antigen (HLA) haplotypes and genetic variations have been linked to DRESS risk. For example, for certain at-risk ethnic populations, there is an association of HLA gene polymorphisms with DRESS for certain medications [19]. Also, polymorphisms in genes encoding drug metabolism enzymes (e.g., cytochrome P (CYP) 450) may cause the medication or its active metabolites to accumulate, increasing the risk of hypersensitivity responses [20]. Furthermore, there is evidence that the risk of DRESS for specific medications varies with dosage. For instance, genetic predisposition and renal impairment work in concert to increase the risk of allopurinol-induced DRESS, which is evident in this case [21]. 

Although the pathogenesis of DRESS is not fully understood, it is considered a T cell-mediated hypersensitivity reaction. Two pathogenic mechanisms are involved in the development of DRESS; drug-specific immune response, demonstrated in vitro by the production of large amounts of tumor necrosis factor (TNF)-alpha and interferon (IFN)-gamma by drug-specific CD4+ and CD8+ T cells, as well as by the positive patch test results for some of the causative drugs [22,23], and reactivation of *Herpesviridae*, a commonly recognized DRESS-related phenomenon. Reactivation of *Herpesviridae* occurs in up to 75% of patients. Human herpesvirus 6 (HHV-6) is the most common virus in the family linked with DRESS and its reactivation is documented in 16-60% of cases [24]. 

DRESS syndrome exhibits a wide range of clinical presentations. The onset of clinical manifestations is not immediate, and typically occurs two to eight weeks following the introduction of the trigger medication [7]. Common symptoms include fever, rash, lymphadenopathy, hematological abnormalities (leukocytosis, eosinophilia, etc.), and abnormal liver function tests that can mimic viral hepatitis [25]. 

The urticarial, maculopapular eruption is the most common cutaneous symptom; vesicles, bullae, pustules, purpura, target lesions, facial edema, cheilitis, and erythroderma can also occur [6]. Occasionally, eosinophilia might not be present, thus making the diagnosis more difficult [10]. 

Visceral involvement involves (hepatitis, pneumonitis, myocarditis, pericarditis, nephritis, and colitis) [10]. Liver injury is the most frequent visceral manifestation of DRESS, occurring in 53-90% of patients [26]. Hepatocellular (19%), mixed (27%), and cholestatic (37%) are the three types of liver injury patterns. Acute liver failure is uncommon and may necessitate liver transplantation, but mostly, abnormalities in liver function tests are mild and transient [27]. 

The spectrum of DRESS-induced kidney injury ranges from proteinuria to renal failure. Acute interstitial nephritis can occur in up to 10-30% of DRESS cases; acute renal failure can occur in up to 8% of patients, and 3% of patients will require either short-term or long-term dialysis [26]. Older age and underlying renal or cardiovascular illnesses are risk factors for the development of drug-induced kidney damage, and renal involvement is also more common with DRESS cases associated with allopurinol [28].

The clinical course of DRESS is variable. Cutaneous and visceral involvement generally resolve gradually after drug withdrawal [29]; however, 25% of patients suffer from flare-ups [30]. DRESS syndrome has a significant risk of fatality, with various studies estimating that mortality rates are in the range of 10% [31]. Diagnosis of DRESS syndrome is mainly clinical; latency period and symptom diversity should be considered. The most popular criteria for validating or rejecting a DRESS diagnosis are included in the Registry of Severe Cutaneous Adverse Reactions (RegiSCAR) scoring system [18]. 

Management of DRESS syndrome depends on prompt recognition of the case and withdrawal of the offending drug. Treatment is usually symptomatic and supportive. Corticosteroids can be used in severe cases and with lung involvement. Other second-line therapies include cyclosporin, intravenous immunoglobulins (IVIGs), and other immunosuppressive agents [10]. 

Allopurinol is a hypoxanthine analog and potent inhibitor of the xanthine oxidase enzyme that is responsible for forming uric acid in the breakdown pathway of purines; hence, allopurinol is a common treatment for hyperuricemia. Allopurinol is associated with a unique form of liver injury that is accompanied by evident immunoallergic manifestations and is a potent trigger of DRESS syndrome [32]. 

Multiple cases have observed the adverse effects of allopurinol. Yaylacı et al. report a case of a male patient who had a similar presentation as the current case with jaundice, generalized maculopapular, and elevated liver enzymes as a result of allopurinol used to treat gout. The patient also expired due to multiple organ failure [33]. Raper et al. report another case with DRESS syndrome and acute liver failure due to allopurinol which was complicated by sepsis and multiorgan failure [34]. Karthika et al. reported a case of a patient who suffered from itching and rash due to allopurinol and who improved after topical steroid and withdrawal of allopurinol [35]. Chaudrey et al. also reported a case of allopurinol-induced DRESS syndrome who presented with elevated liver enzymes and improved after withdrawal of allopurinol which exempted the need for steroid therapy [36]. Another case of allopurinol-induced DRESS syndrome was reported by Yang et al. in which the patient suffered from recurrent attacks of elevated liver enzymes and rash despite steroid therapy [37]. 

Drug use needs to be thoroughly investigated in patients who have complaints of jaundice, generalized rash, acute renal failure, and acute liver failure. Prudent use of allopurinol may reduce the prevalence and morbidity associated with DRESS syndrome. 

## Conclusions

DRESS syndrome is a rare and potentially life-threatening condition with variable presentations that poses a diagnostic difficulty. A high degree of clinical suspicion is needed for prompt diagnosis and management. Caution and observation should be practiced while administering known causative drugs. We present a rare case of severe DRESS with multiple visceral involvements associated with allopurinol. The insidious onset and atypical presentation of the case made it difficult to find a correct diagnosis. Immediate withdrawal of the causative drug is essential to avoid fatal outcomes in DRESS syndrome. Our case highlights the detrimental side effects of allopurinol on the liver and kidney functions of susceptible individuals. 
